# Preventing Age-Related Nuclear Cataract Development – Is Cholesterol the Key?

**DOI:** 10.1167/iovs.67.1.34

**Published:** 2026-01-15

**Authors:** Witold Karol Subczynski, Laxman Mainali, Ross Frederick Collery, Marta Pasenkiewicz-Gierula, Justyna Widomska

**Affiliations:** 1 Department of Biophysics, Medical College of Wisconsin, Milwaukee, Wisconsin, United States; 2 Department of Physics, Boise State University, Boise, Idaho, United States; 3 Biomolecular Sciences Graduate Programs, Boise State University, Boise, Idaho, United States; 4 Department of Ophthalmology and Visual Sciences, Medical College ofWisconsin, Milwaukee, Wisconsin, United States; 5 Department of Computational Biophysics and Bioinformatics, Jagiellonian University, Krakow, Poland; 6 Department of Biophysics, Medical University of Lublin, Lublin, Poland

**Keywords:** eye lens, cholesterol, α-crystallin, cholesterol functions, age-related cataract, protection mechanisms

## Abstract

The cholesterol content in the membranes of the fiber cells of the human eye lens is significantly higher than in any other cell of the body. This review examines the existing literature on the origin and function of this unique feature as one of multiple factors that may help protect against age-related cataract formation throughout a person's life. Three independent sets of experimental data are highly suggestive that high cholesterol content in the fiber cell membranes may protect against cataract formation during aging: (1) saturating cholesterol content preserves the physical properties of the lipid bilayer of the lens cell membranes when the lipid composition of the bilayer changes; (2) high cholesterol content hinders the binding of cytoplasmic α-crystallin to the lipid membrane, which reduces light scattering; and (3) genetic upregulation of cholesterol biogenesis in zebrafish lenses protects against cataract formation in predisposed mutants, whereas administration of cholesterol-lowering statins cause cataracts to reappear.Understanding why the lens contains such high levels of cholesterol is essential for describing its fundamental biology, determining how environmental and genetic factors impact its transparency, and developing treatments for lens opacities. As humans age and are repeatedly exposed to oxidative stress and environmental damage, it is crucial for both researchers and clinicians to comprehend the mechanisms that protect against cataract formation.

Studies on cataracts have led to the conclusion that age is the major risk factor for development of cataract.^1^^–^^3^ Cataracts are caused by age-related changes in the eye lens that gradually accumulate and become apparent when one gets older.^3^ The changes are a consequence of oxidative stress,^4^^,^^5^ α-crystallin membrane binding,^6^^–^^12^ and development of a barrier to nutrient diffusion to the lens center.^13^^,^^14^ Other, less age-dependent factors may also predispose the lens to cataract formation. They include disturbance of oxygen regulation^15^^–^^19^ and disturbance of cholesterol (Chol) metabolism^20^^,^^21^ and defects in lens circulation processes.^22^^–^^24^ One should thus look at cataracts as a multifactorial disease^25^^,^^26^ where several factors play a role.

The lens of the eye consists of fiber cells arranged primarily in concentric layers. Fiber cells differentiate from lens epithelial cells and degrade all cytoplasmic organelles during terminal differentiation. The outer lens fibers form the lens cortex. The central lens fibers form the lens nucleus.^27^^–^^29^ Chol is a natural component of human cell membranes. Its content in a typical plasma membrane ranges between 10 and 30 mol%, that is, the cholesterol/phospholipid (Chol/PL) molar ratio is between 1:9 and 1:2.^30^ In the membranes of certain cells, such as red blood and Schwann cells, this content is significantly higher, close to 50 mol%, that is, the Chol/PL molar ratio is close to 1:1.^31^^,^^32^ In the membranes of the fiber cells of the human eye lens, Chol levels are even higher.^33^ This high Chol content in these membranes is likely an essential factor in preventing cataracts.

The aim of this paper is to propose how Chol may counteract the degenerative processes that occur in the eye lens throughout a person’s lifespan and lead to age-related cataracts and impaired vision. Investigations of the physicochemical and structural properties of lipid bilayers that model the lipid matrix of the membranes of lens cells and of intact membranes have yielded three major findings that allow formation of a hypothesis that Chol is key to the preservation of lens transparency.

First, the Chol content in the fiber cell membranes is extremally high,^33^ much higher than in any other cell membrane in the human body.^30^ In all lenses examined, Chol content is at least saturating, for example, the Chol/PL molar ratio is 1.^34^^,^^35^ When the ratio exceeds 1, pure Chol bilayer domains (CBDs) form within the membrane.^36^^,^^37^ CBDs serve as Chol reservoirs and ensure that the surrounding lipid bilayer (PL-Chol domain) is saturated with Chol. With age, Chol content in the fiber cell membranes increases,^38^ reaching a total Chol/PL molar ratio as high as 4.^33^^,^^39^^–^^41^ However, not all of this Chol then accommodates in the membrane; some, in the form of Chol crystals, is most likely detached from it. As discussed in a subsequent section, the saturating Chol content in the fiber cell membranes of a transparent lens is needed to maintain the homeostasis of the membranes, fiber cells, and eye lens itself. This multi-level homeostasis is essential to preserve lens transparency.^21^^,^^37^^,^^42^

Second, another finding concerns α-crystallin, a water-soluble structural protein found in the cytoplasm of lens fiber cells. It exists in the cytoplasm as polydisperse mixtures of 24 to 28-mers,^43^ which play a major role in maintaining lens transparency. With age, a fraction of α-crystallin in the lens cell cytoplasm decreases,^6^^–^^8^^,^^11^ whereas the cell membrane-bound α-crystallin increases.^11^^,^^12^^,^^44^^,^^45^ This leads to blurred vision and contributes to cataract development. Model studies carried out on single-lipid bilayers made of major PLs of the fiber cell membranes phosphatidylcholine (PC), phosphatidylserine (PS), phosphatidylethanolamine (PE), and sphingomyelin (SM)^46^ and on mixtures of these PLs^47^^–^^49^ show that α-crystallin binds efficiently to all of them. However, when these lipid bilayers contain a saturating concentration of Chol, the binding of α-crystallin is inhibited.^50^^,^^51^ This suggests that Chol protects the cell membrane from α-crystallin binding, and thus from compromising lens transparency and development of cataract.

In Section 4 entitled “Congenital Cataract and Chol,” data dealing with the in vivo studies of the manipulation of Chol which alters progression of cataracts are included. We focused our attention on the in vivo zebrafish mutant model, which we are planning to investigate in detail in the future. These planned experiments should strengthen the evidence about the significant function of Chol in preventing lens cataracts, as well as a deeper exploration of mechanisms of this protective effect. Experiments on a zebrafish mutant model with inactivated α-crystallin (*cryaba–*/–) give further evidence that Chol likely plays a role in preventing cataracts. The mutation in this zebrafish model is associated with lens defects in the form of congenital cataracts accompanied by increased light scattering.^52^^,^^53^ Further genetic modification by mutating Nrf2 (*nfe2l2a–*/*–*), that is, the master oxidative stress factor, is associated with reduction in lens defects.^54^ A detailed analysis shows that several genes responsible for the Chol biogenesis are upregulated in this double-mutant model.^54^ Significantly, administration of statins to this double-mutant model shows re-emergence of cataractous lens defects.^54^ This indicates that Chol metabolism dysregulation is a significant cataract risk factor, and that maintaining Chol balance might aid in preventing or delaying cataract development. However, specific interventions focused on lipid irregularities require additional investigation. On the basis of the experimental findings, we propose a hypothesis that Chol homeostasis is crucial for maintenance of lens clarity.

## Chol Effects on Lipid Bilayers, Lens Lipid Membranes, and Fiber Cell Membranes

### An Overview of Methods of Assessing the Effect of Chol on the Properties and Organization of Lipid Bilayers

**Figure 1.**
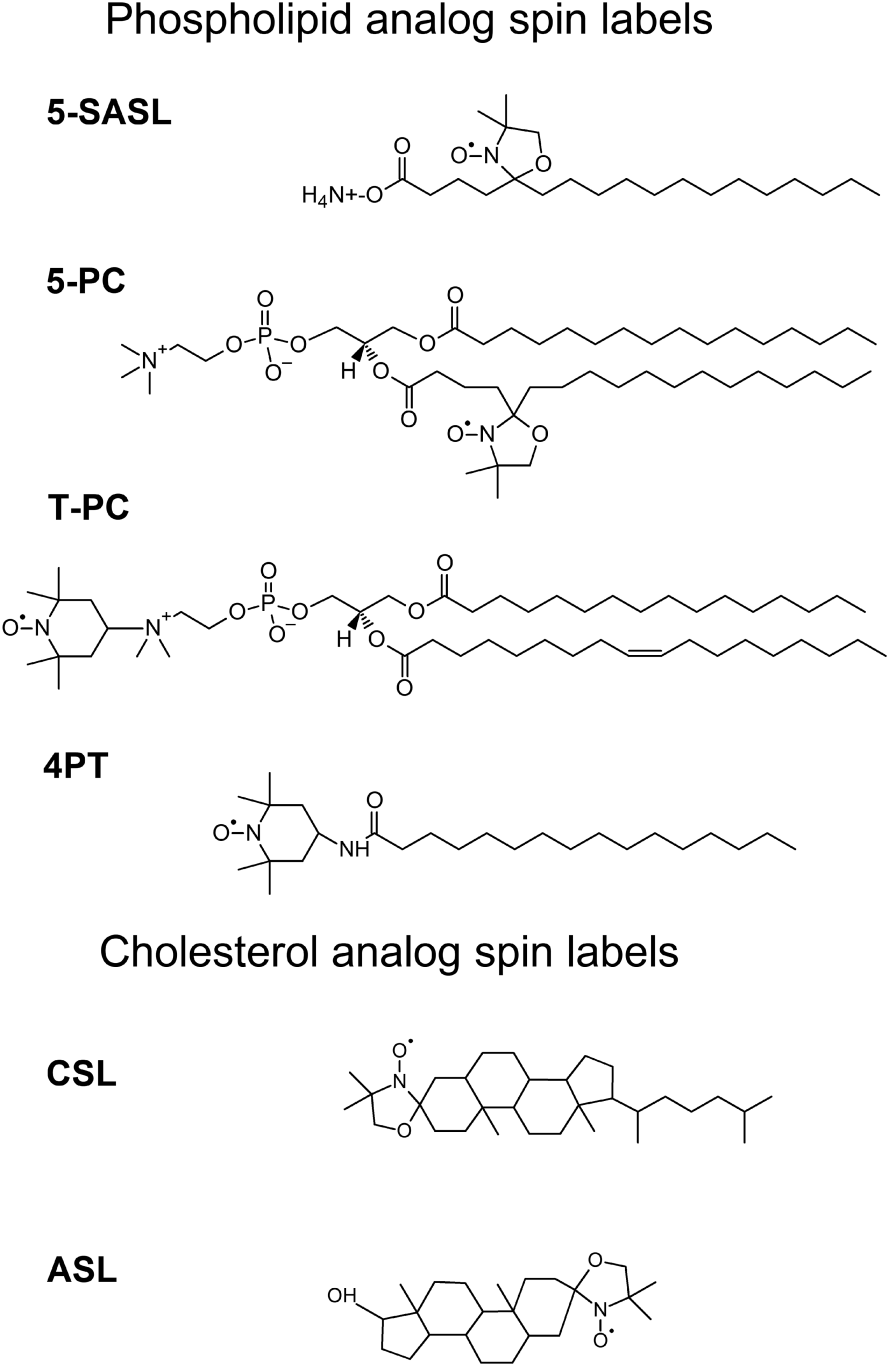
Chemical structures of the representative spin labels from three classes of lipid spin labels used in membrane studies. Stearic acid spin labels: *n*-SASL—n-doxylstearic acid spin label, *n* = 5, 7, 9, 10, 12, 14, and 16. PL spin labels: n-PC—1-palmitoyl-2-(n-doxylstearoyl, *n* = 5, 7, 10, 12, 14, and 16) phosphatidylcholine). 4PT—4-palmitamido-TEMPO; and T-PC—tempocholine-1-palmitoyl-2-oleoylphosphatidic acid ester. Chol-analog spin labels: ASL—androstane spin label and CSL—cholestane spin label.

Third, development of a barrier to nutrient diffusion also influences cataract formation.^13^^,^^55^^,^^56^ Each cell is surrounded by a plasma membrane, whose main structural element is the lipid bilayer. This lipid bilayer acts as a barrier, preventing uncontrolled transport of material into and out of the cell, regulating the bulk properties of the membrane, providing a natural environment for integral membrane proteins, and supporting membrane protein function. In contrast, membrane proteins control transportation and communication between the interior and exterior of the cell. They selectively locate, function, and assemble in membrane domains with specific properties. Chol is essential for the formation of these domains. Information on how Chol affects the lateral organization of a PL bilayer, particularly information on how Chol induces formation of domains and separate phases, can be obtained from phase diagrams. A phase diagram shows the limits of stability of the various phases in a system at equilibrium, with respect to temperature and composition^21^^,^^57^ but does not provide information about the structure and physicochemical properties of the phases and domains. This information can be obtained from conventional and saturation-recovery (SR) electron paramagnetic resonance (EPR) measurements.^58^^–^^60^ To use these techniques, spin-labeled (SL) lipids (lipid spin labels) must be introduced to the bilayer. An SL lipid is a modified PL or Chol molecule that has a nitroxide radical covalently attached to one of its carbon atoms. The nitroxide plays the role of a monitoring group (see Fig. 1 for structures of typical lipid spin labels). Because the distributions of SL and unlabeled lipids in the bilayer are expected to be similar, the lipid SLs give a reliable account of the local properties of the membrane. In practice, to some extent the nitroxide group perturbs the neighboring lipids but because the labeled lipids are well diluted in the bilayer (the usual labeled-to-unlabeled lipid molar ratio in the bilayer is 1:100), the overall effect of this perturbation is rather small. In the SL-PL, the nitroxide is attached to either the polar head group or a chosen carbon atom along the acyl chain (see Fig. 1). In the SL Chol (SL-Chol), the label replaces the OH group (cholestane and androstane), whereas in androstane, an additional OH group replaces the Chol hydrocarbon chain (see Fig. 1). Lipid spin labels can thus report on the properties of the bulk membrane and each of the membrane phases or domains at various depths. This enables us to obtain a near-complete three-dimensional representation of the membrane properties, and thus detailed information about the lateral and vertical organization of the membrane.

**Figure 2.**
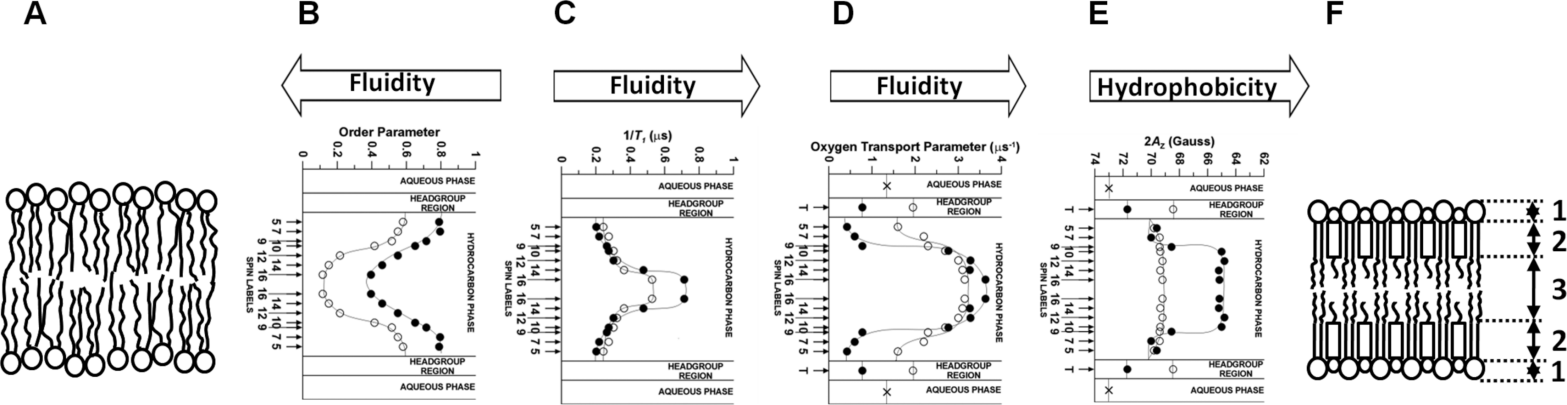
Profiles of different membrane properties across egg sphingomyelin bilayers in the absence (°) and presence (●) of a saturating amount (50 mol%) Chol. All profiles were obtained at 37°C with the PL-analog spin labels (n-PC, 9-SASL, and T-PC) and symmetrized for both bilayer leaflets (see Supplement where measured values are explained). Data points were taken from References 73 and 75. Order parameter (**B**), which describes the angular amplitude of the wobbling of the acyl chain segment, and membrane fluidity. (**C**), measured as spin-lattice relaxation rate, *T*_1_^−1^ (which is also the measure of the rate of spin label rotational diffusion^79^), and oxygen diffusion-concentration product (**D**) (oxygen transport parameter, OTP) are dynamic parameters, Hydrophobicity (**E**) measured as the hyperfine splitting 2*A*_Z,_ are static parameters. Approximate locations of the nitroxide moieties of the spin labels are indicated by arrows (numbers for n-PC, 9-SASL, and ‘T’ for T-PC). Schematic drawings of the PL bilayer membranes without (**A**) and saturated with Chol (**F**). The different effects of Chol at different membrane depths are summarized on the right side. The five distinct regions across the entire membrane saturated with Chol can be clearly visualized: (1) headgroup region; (2) ordered and condensed region; and (3) fluid membrane center. It is apparent that saturation with Chol smooths the surface of the bilayer.^80^ Because of the Chol condensing effect the thickness of the bilayer saturated with Chol is greater than that of the bilayer without Chol. Here, to make the comparison easier, the profiles were adjusted by the localization of the nitroxide moieties of spin labels in both bilayers.

Because this review concerns the eye lens, and sphingolipids constitute 66% of all phospholipids in the fiber cell membrane of an adult human lens,^61^ an SM bilayer is used here as a first simple model of the membrane. As explained above, SL EPR spectroscopy can provide depth-dependent properties of the membrane by using SL PLs. Static properties of the membrane, such as ordering^62^ and hydrophobicity^63^^–^^65^ (refer to the Fig. 2 legend and Supplement for explanations), are directly obtained from the SL EPR spectra. Motional parameters, such as the spin-lattice relaxation rate (*T*_1_^−1^) and the oxygen transport parameter (OTP; see Supplement for explanation), are obtained from SR EPR measurements. *T*_1_^−1^ is highly sensitive to the rotational motion of the nitroxide moiety attached to the lipid^66^^–^^68^; OTP is sensitive to the collisions of the nitroxide with apolar molecular oxygen and informs about membrane fluidity.^60^^,^^69^^,^^70^ Profiles of the order parameter, *T*_1_^−1^, OTP, and hydrophobicity across the SM bilayer are presented in Figures 2B, 2C, 2D, and 2E, respectively. The effect of the saturating amount of Chol on the profiles of the four parameters is clearly visible in Figures 2B to 2E. Figure 2 also includes schematic drawings of the structure of a PL bilayer without Chol (see Fig. 2A) and with a saturating amount of Chol (see Fig. 2F).

The most important result from these model studies is that differences in the four profiles obtained for the studied lipid bilayers, which were significant when the bilayers did not contain Chol, practically disappeared when the bilayers contained the saturating Chol content. This effect of Chol was observed not only for single-PL bilayers^69^^,^^71^^–^^76^ but also for mixed-PL bilayers.^35^^,^^38^^,^^59^^,^^77^ For a more detailed description of the effects of Chol on the properties and organization of lipid bilayers, see the review by Subczynski et al., 2017.^78^

### An Overview of the Effects of Chol on the Properties and Organization of Lens Lipid Membranes

**Figure 3.**
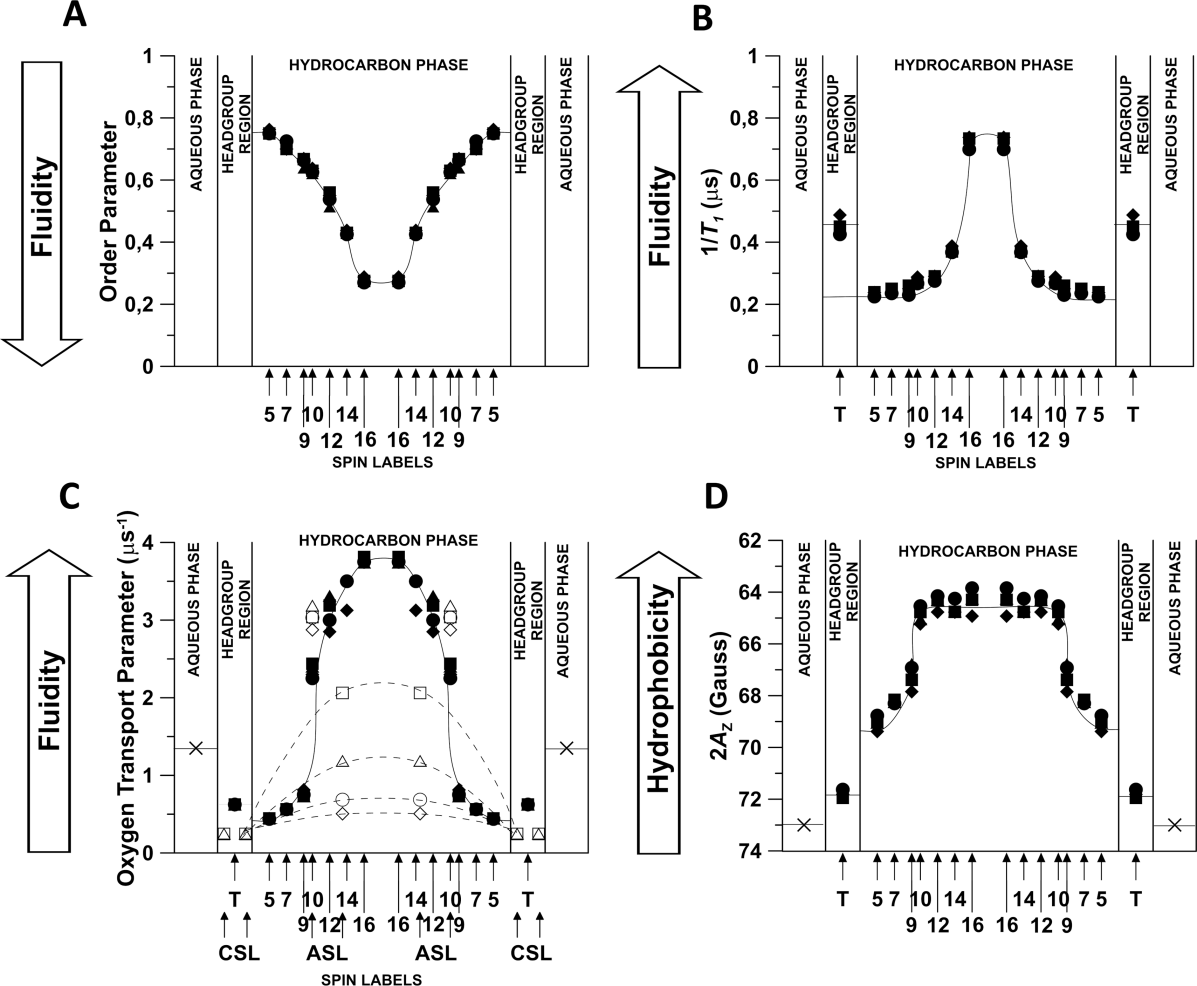
Profiles of different membrane properties across the cortical lens lipid membranes of eye lenses from human donors of different age groups obtained using the EPR spin labeling approaches (see Supplement where measured values are explained). Symbols indicate the age group: ■, □ (0–20 years); ▲, ∆ (21–40 years); ●, ○ (41–60 years); ◆, ♦ (61–70 years). Filled symbols indicate profiles obtained with n-PC and 9-SASL, and open symbols indicate data obtained with ASL and CSL. All profiles were obtained at 37°C for samples from pools of at least 17 clear lenses from donors of each age group. Data obtained with Chol-analog spin labels are included only into the profiles of the OTP. Approximate localizations of the nitroxide moieties of spin labels are indicated by *arrows*. Localizations of the nitroxide moieties of ASL and CSL in the PL bilayer and the CBD are explained in Reference 88. As indicated by profiles of the OTP (data obtained with ASL and CSL), the CBDs are present in membranes of all age groups, indicating that the surrounding PL bilayer is always saturated with Chol. This figure is taken from Reference 89 without any modification (Copyright 2025) with permission from Elsevier.

A more accurate model of the fiber cell membrane than a SM bilayer is a lipid bilayer made from the total lipid extracts of fiber cell membranes called lens lipid membranes (LLMs). The LLMs consist of both PLs and Chol. Investigating the organization and properties of LLMs derived from lenses of donors of different age groups reveals significant age-related changes in the PL composition of the human eye lens membrane.^14^^,^^81^^–^^84^ The content of sphingolipids, including dihydrosphingomyelins and SMs, in the membrane increases with age, whereas that of glycerophospholipid (phosphatidylcholine and phosphatidylethanolamine) decreases.^81^^–^^83^^,^^85^^,^^86^ In young lens donors, sphingolipids constitute approximately 1/3 (approximately 33%) of membrane phospholipids, whereas in older lens donors, approximately 2/3 (approximately 66%) of phospholipids are sphingolipids. Sphingolipids, especially dihydrosphingomyelins, are more saturated than glycerophospholipids and more resistant to oxidation. These changes in the lipid composition affect the physical properties of the lipid matrix of fiber cell membranes and the functioning of integral membrane proteins,^87^ which can lead to developing age-related cataracts. However, aged lenses often function properly with no signs of age-related cataracts. An explanation of why this is possible is included in the profiles of the order parameter, *T*_1_^−1^, OTP, and hydrophobicity across the LLMs obtained from the fiber cells of human lens donors belonging to different age groups, shown in Figure 3.

On the basis of the results presented in Figure 3, it can be seen that independent of the drastic changes in PL composition that occur in the fiber cell membranes with age, their over-saturating Chol content ensures that the bulk physical properties of these membranes are the same. Profiles obtained from the EPR spectra of the Chol-analog spin labels, ASL and CSL to check the location of ASL and CSL in the PL bilayer and the CBD see Reference 88, indicate the CBDs are present in the membranes of all age groups, including the youngest human donors (see Fig. 3C). They show that the PL-Chol bilayer domain that surrounds CBDs is always saturated with Chol. This, in turn, is confirmed by the profile obtained from the EPR spectra of the PL-analog spin-labels, SL-PL (see Fig. 3C). This figure, which shows profiles of the OTP, suggests that Chol saturation is not an age-related adaptation and that Chol saturation of fiber cell membranes occurs at the early stages of fiber cell maturation. We further discuss this phenomenon in Section 2.3 entitled “An Overview of the Effects of Chol on the Properties and Organization of the Membranes of Fiber Cells.”

The majority of published data focuses on the Chol composition of healthy transparent lenses.^35^^,^^38^^,^^89^ Data about Chol content in cataractous lenses are more sparse and scattered. Data obtained for lens membranes prepared with the rapid solvent exchange method,^90^^,^^91^ which protect samples from the artifact formation of Chol crystals, indicate that Chol/PL ratio in cataractous lenses is significantly lower than in transparent lenses.^34^ The Chol/PL ratio in cortical and nuclear fiber cells of 61 to 70-year-old human transparent lenses was 1.8 and 4.4,^34^ respectively, whereas this ratio in cortical and nuclear membrane for age-matched cataractous lenses was 1.14 and 1.45,^34^ respectively. The high Chol content in clear nuclear membranes induced formation of Chol crystals (which are not observed in cataractous nuclear membranes). These results agree with other reports that Chol content in cataractous lenses is lower than in clear lenses.^92^ However, some older papers indicate opposite trends.^93^^,^^94^

By interacting with phospholipids and proteins, Chol plays a crucial function in regulating the physico-chemical characteristics of cell membranes. Saturating Chol content in the lens membranes increases their stiffness, which contributes to presbyopia. As shown on model membranes, the mechanical rigidity of the PL bilayers (rigidity to macroscopic deformation like bending) has been shown to increase with the Chol concentration.^95^^–^^97^ Most significantly, however, is the fact that Chol increases the mechanical rigidity of the sphingolipid bilayer approximately one order of magnitude higher than for bilayers from other three PLs present in fiber cell membranes, namely PS, PE, and PS.^95^ It is known that the SM content in human fiber cell membranes increases until the age of approximately 50 years, the age when presbyopia starts to appear. This correlation has not yet been explored but strongly suggests that increasing Chol and SM-associated increase of membrane stiffness may be one of the mechanism responsible for presbyopia.

### An Overview of the Effects of Chol on the Properties and Organization of the Membranes of Fiber Cells

The Chol content of fiber cell membranes of the eye lens depends on the maturity of the lens and is at least 50 mol%.^39^ Additionally, fiber cell membranes are rich in sphingomyelins.^61^ Because raft domains are defined as rich in Chol and sphingolipids that form small heterogeneous domains,^92^ it would seem that raft-domains should play significant role in fiber cell membrane functioning. However, the unique composition of lens fiber cell membranes does not allow for the formation of heterogeneous raft domains, which are used for varied processes in other cell types such as cell-cell signaling, and molecular sorting and trafficking. Even in young lenses, the fiber cell membranes are saturated with Chol which is ensured by the presence of CBDs. The lipid bilayer of such a membrane is in the liquid-ordered phase that is characteristic of the lipid raft.^98^ This makes the bilayer of a young fiber cell a nearly uniform, homogeneous raft-like domain, in which integral membrane proteins are embedded. This phase state renders the bilayer impartial to the changes in the PL composition (see Fig. 3) and ensures proper functioning of the membrane proteins, despite any significant age-related changes in the membrane lipids. In older lenses, the increased Chol content in the membrane enhances the formation of CBDs. However, they do not affect either the Chol-saturated PL bilayer or the embedded integral membrane proteins.

Because mature fiber cells of the eye lens are metabolically inactive,^99^ communication and transport between the cells proceeds via an extensive network of cell-to-cell junctions, of both gap junction and thin junction types.^100^ Thin junctions are similar to tight junctions seen in other cell types, but serve to maintain cell adhesion rather than sealing the space between cells. Gap junctions are clusters of membrane channels that are permeable to water, ions, and small molecules. Lens gap junctions in fiber cells are formed predominantly by two connexins, Cx46 and Cx50.^22^^,^^101^ Six connexins form a connexon, which is a half-channel. The connection between two connexons from adjacent cells creates an intercellular channel, that is, a gap junction. Another transmembrane protein, aquaporin-0 (AQP0) is a water channel expressed almost exclusively in fiber cells of the eye lens.^102^ The coupling of two tetramers of AQP0 from neighboring fiber cells makes a thin junction.^103^

**Figure 4.**
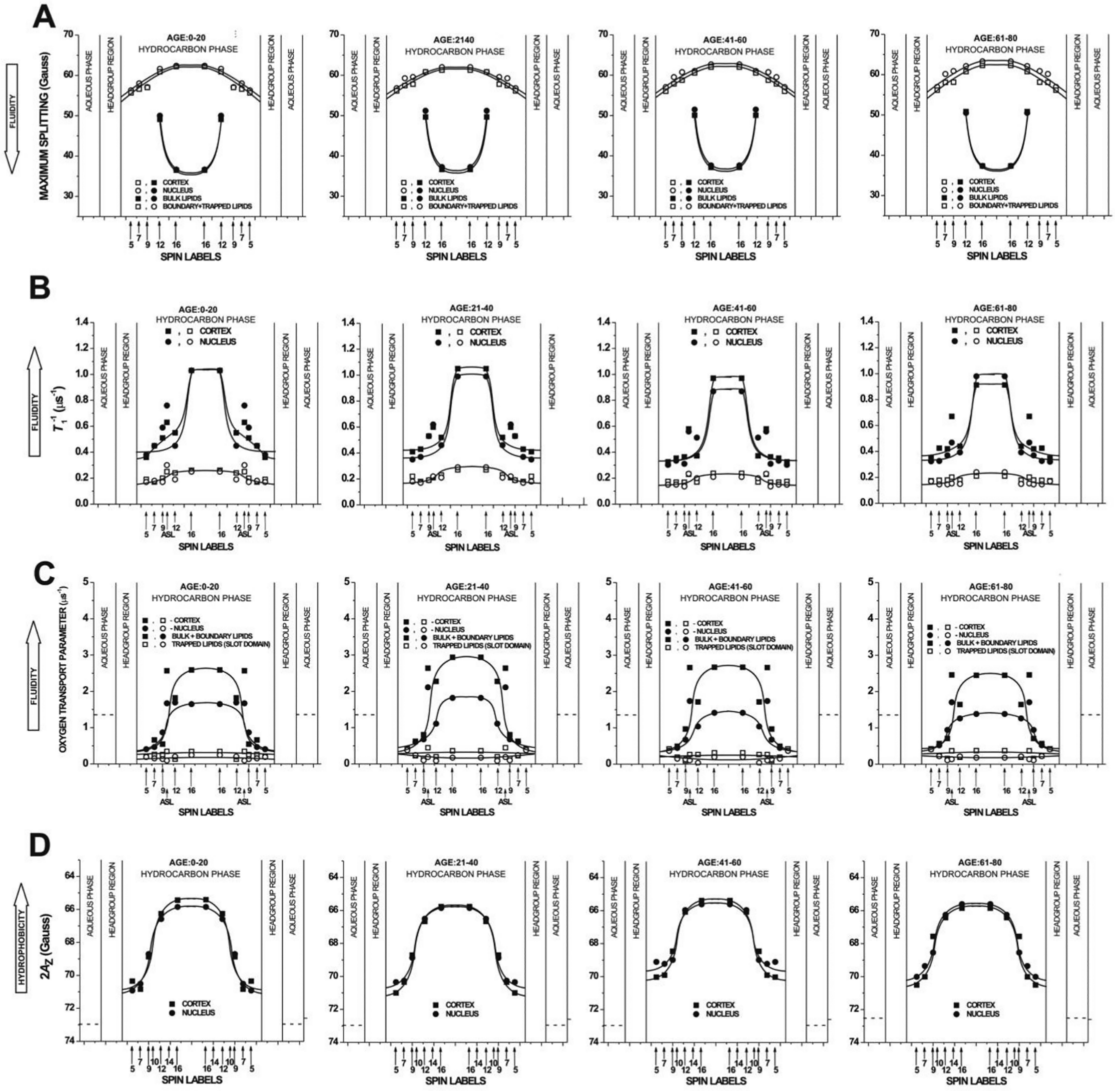
Profiles of different membrane properties across intact cortical (■, □) and nuclear (●, ○) fiber-cell plasma membranes of eye lenses (see Supplement where measured values are explained). Profiles were obtained using EPR spin-labeling methods with n-SASLs at 37°C for samples from pools of approximately 20 clear lenses from donors of four age groups (0–20 years, 21–40 years, 41–60 years, and 61–80 years). Profiles of the maximum splitting (**A**) are reported for bulk lipids (■, ●) and for boundary plus trapped lipids (□, ○). Profiles of the *T*_1_^−1^ (spin-lattice relaxation rate) (**B**) are reported for bulk plus boundary lipids (■, ●) and for trapped lipids (□, ○). The values of *T*_1_^−1^ obtained with ASL in domains of cortical and nuclear membranes are also included. Profiles of the OTP (**C**) are reported for bulk plus boundary lipids (■, ●) and for trapped lipids (□, ○). The values obtained with ASL in domains of cortical and nuclear membranes are also included. Hydrophobicity profiles (2*A*_Z_) (**D**) represent averaged values from bulk, boundary, and trapped lipids (■, ●). Approximate localizations of the nitroxide moieties of spin labels are indicated by *arrows*. This figure is taken from Reference 104 without any modification (Copyright 2025) with permission from Elsevier.

Membrane proteins and lipid bilayers in which they are embedded mutually influence each other. The effect of membrane proteins on the properties of the membrane is clearly visible in the profiles of the maximum splitting, spin-lattice relaxation rate (*T*_1_^−1^), OTP, and hydrophobicity across different domains in the intact plasma membranes of fiber cells, displayed in Figure 4 (and further elaborated in the Supplementary material). The differences between LLM and an intact membrane is that LLM is the lipid fraction of the fiber cell membrane only, whereas the intact membrane also contains membrane proteins embedded in its lipid bilayer. Integral membrane proteins induce formation of three distinct lipid domains in the bilayer; namely bulk lipid, boundary lipid, and trapped lipid domains. The bulk lipid domain is the least affected by the presence of integral proteins. This domain can be discriminated from the boundary and trapped lipid domains by using the maximum splitting as a parameter. Maximum splitting refers to the distance between the outermost lines in the EPR spectrum, measured in magnetic field units (Gauss). The profiles of maximum splitting across different membrane domains are illustrated in Figure 4A. The profiles of *T_1_^−1^* shown in Figure 4B and the OTP shown in Figure 4C can differentiate trapped lipids from bulk and boundary lipid domains. The profiles of averaged membrane hydrophobicity are shown in Figure 4D. Membrane hydrophobicity is assessed from an EPR spectrum of frozen membrane suspension; therefore, it is not possible to differentiate the domains from the spectrum. It is important to note that all profiles presented in Figure 4 were derived from the SL-PL EPR spectra, and SL-PLs do not partition into CBDs. Thus, the results are largely unaffected by the presence of CBDs in the membrane.

Results presented in Figure 4C indicate the presence of CBDs in intact membranes isolated from lenses of donors of different ages (including an age group of 0–20-year-old donors).^104^ Because the CBDs form the buffering capacity for Chol molecules in the surrounding PL bilayers, we can assume that these bilayers in membranes, already undergoing fiber cell maturation, are saturated with Chol. However, the Chol content in fiber cell membranes and in fiber cells themselves increases with age beyond the saturation level that forms CBDs and Chol crystals. Surprisingly, values of Chol saturation limits (after which CBDs are formed) and Chol solubility thresholds (after which Chol crystals are formed) in practice do not change with age independently of the drastic changes in the PL composition (see the appropriate explanation in the Supplement and the data presented in the Supplementary Fig. S1). Mature fiber cells do not contain intracellular organelles, including inflammasomes. Additionally, the lens is avascular thus protected against the inflammatory cascade. Formation of CBDs and even Chol crystals do not induce inflammation and do not affect lens transparency. This is highly contrasted with membrane responses of other tissues and organs when Chol levels go up. When the Chol content in membranes of other tissues and organs increase above the Chol solubility threshold and Chol crystals start to form, this is taken to be a sign of a pathological condition.^105^ For instance, it was shown that the deposition of minute Chol crystals in arterial cells initiates and promotes atherosclerosis.^106^^–^^108^

### Chol Maintains Homeostasis of Lens Membranes, Fiber Cells, and Eye Lens

#### Membrane Homeostasis

The main function of the eye lens is to focus light onto the retina. The lens must remain transparent throughout a person’s life. The lens remains transparent when specific conditions at the level of the fiber cell, its membrane, and its cytoplasm are met. The first condition is that the lens is free of cytoplasmic organelles, such as the nucleus, endoplasmic reticulum, and mitochondria, which scatter light.^109^ The second condition is a high Chol content in the cell membrane. Because the lens lacks a vascular system, Chol cannot be delivered to the cell membrane from plasma lipoproteins.^99^ Instead, its availability depends exclusively on de novo Chol biosynthesis in the lens epithelium.^110^

Because the lens is avascular, its PLs and Chol level is independent of diet.^111^ It has been reported that synthesis and remodeling of the lens’ own lipids^41^^,^^111^ including Chol,^20^^,^^111^^–^^114^ can occur in situ within the lens itself. This process ensures a high membrane Chol content in cortical and nuclear membranes necessary to help the human lens be transparent throughout the entire human life, which approaches a century in the modern era. This additionally assures that the physicochemical properties of the membranes are constant, which is vital for the proteins embedded in the membranes. Because there is no protein turnover in mature fiber cells,^115^^,^^116^ the activities of the membrane and cytoplasmic proteins must remain consistent at any lens age. This is seeming in conflict with the changes in membrane PL composition that occur during aging.^39^^,^^81^^,^^85^^,^^117^^,^^118^ However, as discussed in Section 2.3, high Chol content and the presence of CBDs in the membranes of fiber cells ensure that the PL-Chol membrane domain is saturated with Chol. This helps to maintain the homeostasis of the membranes and cells during age-related changes in their PL composition.

#### Fiber Cell Homeostasis

As described in Section 2.3, AQP0 and the connexins Cx46 and Cx50^119^^–^^123^ are the most abundant proteins found in the membranes of lens fiber cells.^124^ These proteins form thin junctions and gap junctions that facilitate the transport of water and specific solutes, including glucose, amino acids, and polar antioxidants, and also ions in the case of gap junctions.

The high Chol content in the membranes of fiber cells generates a steep hydrophobicity gradient, resulting in a dielectric constant at the membrane's center that is close to that of hexane (see Fig. 3D), regardless of the membrane's PL composition.^75^ This gradient ensures that the circulation of water, ions, and small polar solutes between fiber cells is rigorously controlled, as this occurs exclusively through cell-cell junctions. This is crucial for maintaining the proper functioning of fiber cells. It should be noted that membranes of human fiber cells contain highly saturated PLs,^114^ mainly sphingolipids.^82^^,^^125^ When such a membrane lacks Chol or has a low Chol content, it is permeable to water and small solutes. This is apparent from a relatively flat hydrophobicity profile and a dielectric constant at the membrane's center that is significantly higher than that of hexane (see Fig. 2C).^59^^,^^75^

#### Eye Lens Homeostasis

Oxygen is a factor that damages the transparency of the eye lens, leading to cataracts.^4^ The primary mechanism that protects the lens from becoming opaque is maintaining an extremely low oxygen partial pressure within it. This can be achieved through three processes: (1) low oxygen partial pressure at the lens surface, (2) oxygen consumption within the lens, and (3) barriers to oxygen permeation across layers of fiber cells.

The first two processes do not involve Chol, but the third one does. When the Chol level in the lipid bilayer modeling the membranes of fiber cells is saturating, the oxygen permeability coefficient across this bilayer is significantly lower than that across a similarly thick layer of water.^104^ Additionally, the oxygen permeability coefficient across CBDs, which constitute a large part of the membrane surface, is approximately 10 times smaller than that of the bulk PL-Chol domain surrounding these CBDs.^34^^,^^126^ Consequently, an oversaturating Chol content in the membranes of fiber cells is should act as a barrier to oxygen penetration. It is also important to note that fiber cells are arranged in concentric layers. Although the difference in oxygen partial pressure across an individual cell membrane is small, oxygen must pass through roughly 2000 membranes to travel from the lens surface to the lens center. This arrangement contributes to a significant reduction in oxygen partial pressure in the lens center.

In conclusion, a high Chol content in the membranes of fiber cells ensures (1) their constant physicochemical properties despite changes in the membrane PL composition, which is essential for the proper functioning of membrane proteins; (2) a high hydrophobic barrier across the membrane, which is necessary for the controlled circulations of water and solutes between cells; and (3) a low oxygen partial pressure, which protects the lens from oxidative stress. In this way, the homeostasis of lens membranes, fiber cells, and the lens itself are preserved.

## The Effects of Chol on α-Crystallin Binding to Membranes

### α-Crystallin in the Eye Lens Cytoplasm

The α-crystallin is a water-soluble structural protein of the cytoplasm of fiber cells, making up approximately 35% of the total soluble protein in the lens.^127^ It functions as a molecular chaperone, preventing stress-induced aggregation of other cytoplasmic proteins. The α-crystallin consists of two subunits: αA-crystallin and αB-crystallin. Whereas αA-crystallin is primarily expressed in the lens, αB-crystallin is expressed in other tissues as well. The αA- and αB-isoforms form hetero-oligomers (αA/αB), typically in a 3:1 ratio.^128^^,^^129^ In the cytoplasm, α-crystallins form polydisperse mixtures of soluble 10 to 40-mers, with the most common size of 24 to 28-mers.^43^^,^^127^

Not all cytoplasmic α-crystallin is in a soluble form; some are associated with lens fiber cell membranes. Both forms exhibit chaperone activity, although this activity is weaker in the bound form. In young lenses, there is an equilibrium between membrane-bound and soluble α-crystallin, with the fraction of membrane-bound α-crystallin being significantly smaller. However, as the lens ages, cumulative damage to proteins leads to the formation of large aggregates of α-crystallin and other crystallins. This reduces the soluble α-crystallin fraction and increases the membrane-bound fraction.^9^ Because there is no protein turnover in the lens, the aggregated α-crystallin cannot be replenished or replaced by newly synthesized molecules.^116^^,^^130^ The aggregation of cytoplasmic proteins gives rise to light scattering, reducing lens clarity and diminishing unobstructed vision. Therefore, the solubility, polydispersity, and molecular chaperone function of α-crystallin are crucial for maintaining lens transparency and preventing cataracts.^128^

In older lenses, levels of membrane-bound α-crystallin significantly increase as cataracts develop, compared with levels in younger lenses. In young lenses, metabolites can diffuse freely between the nuclear and cortex cells through gap junctions. However, in older lenses, the accumulation of α-crystallin bound to cell membranes may create a barrier to metabolite diffusion, blocking the flow of small molecules such as polar antioxidants (e.g. glutathione and ascorbate) between the nucleus and cortex.^9^ This barrier to diffusion can induce cataract development.

However, it is not uncommon for the lenses of older individuals to remain transparent with no signs of cataracts. This suggests that there is a mechanism that reduces the binding of α-crystallin to the membranes of fiber cells. The experimental results presented below suggest that this mechanism is associated with a high Chol content in the membranes.

### The Effects of Chol on the Interaction of α-Crystallin With the Four Major PLs of the Eye Lens Membranes

**Figure 5.**
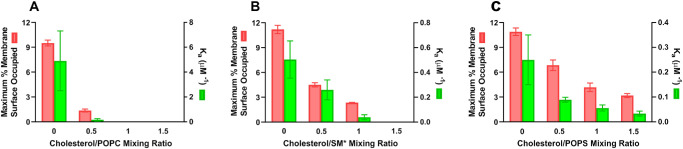
The maximum percentage of membrane surface occupied (MMSO) by α-crystallin and the binding affinity (*K*_a_) of α-crystallin binding to the Chol/PL membranes at Chol/PL mixing ratios of 0, 0.5, 1, and 1.5. (**A**) Chol/POPC membranes; (**B**) Chol/SM* membranes, where * represents the presence of 20 mol% POPS; and (**C**) Chol/POPS membranes. This figure is taken from Reference 131 without any modification and with permission from MDPI.

As mentioned in the introduction, α-crystallin binds effectively to both single-lipid and mixed-lipid bilayers composed of the major PLs of the membranes of human fiber cells (PC, PS, PE, and SM; Fig. 5 is taken from Ref. 131 but see also Refs. 41–44).

Detailed model studies on the interactions between α-crystallin and lipid bilayers composed of the PLs found in the membranes of human fiber cells show that this binding depends on the type of head group and acyl chains of the PLs.^46^^,^^132^^,^^133^ For PLs with palmitoyl-oleoyl acyl chains (i.e. POPS, POPC, and POPE, as well as SM with a palmitoyl chain) the maximal surface area occupied by bound α-crystallin is greatest for SM, followed by PS and PC, whereas there is no binding to POPE. To evaluate how the strength of the binding of α-crystallin to the bilayer depends on the lipid head group, the binding constant, *K_a_*, was measured. The values for *K_a_* are in the order *K_a_* (PC), >*K_a_* (SM), and >*K_a_* (PS; see Fig. 5).^46^ The dependence of the strength of the binding of α-crystallin to the bilayer, *K_a_*, on the length and unsaturation of the PL acyl chains was determined for di-14:0PC, 18:0-18:1PC, di-18:1PC, and 16:0-20:4 PC bilayers. The values for *K_a_* are in the order *K_a_* (di-14:0PC), >*K_a_* (18:0-18:1PC), >*K_a_* (di-18:1PC), and ≈ *K_a_* (16:0-20:4PC).^133^ For saturated bilayers, the value of *K_a_* is approximately 5 times as high as that for monounsaturated bilayers and approximately 50 times as high as that for polyunsaturated bilayers.^133^ Therefore, the intricate balance between the head groups of PLs, the lengths of their acyl chains, and the degree of unsaturation plays a crucial role in regulating the interaction between α-crystallin and PL bilayers.

Because at least the lipid bilayers of the membranes of fiber cells are saturated with Chol, as a next step, the effect of the Chol content on the binding of α-crystallin in the POPS, POPC, POPE, and SM bilayers was investigated, as discussed by Timsina and colleagues.^51^ Chol strongly affects the binding of α-crystallin to PL bilayers but is different for each type of PL comprising the bilayer.^51^ To evaluate the effect of Chol, two previously introduced quantities were measured as a function of the bilayer Chol content: the binding constant, *K_a_*, and the maximum percentage of membrane surface occupied (MMSO) by bound α-crystallin.^47^ As the Chol bilayer content increases, both *K_a_* and MMSO decrease (see Fig. 5). The effect on the parameters is the strongest for the Chol/POPC bilayers. Complete inhibition of α-crystallin binding occurs when the Chol/POPC molar ratio is 1.0 (at 50 mol% Chol in the bilayer; see Fig. 5A). For the Chol/SM bilayers, complete inhibition of binding occurs when the Chol/SM molar ratio is 1.5 (at 60 mol% Chol in the bilayer; see Fig. 5B). For the Chol/POPS bilayers, even at Chol content as high as 60 mol%, the binding of α-crystallin is not completely inhibited. At 60 mol% Chol, the value of *K_a_* drops to 15% and MMSO to approximately 30% of their initial values (see Fig. 5C). For any Chol mol%, the values of both *K_a_* and MMSO are zero for the Chol/POPE bilayer.

The membranes of lens fiber cells contain very high levels of Chol, which increase with aging, and are greater in nuclear than in cortical membranes. At such high Chol contents, CBDs are formed in the membrane. Both the Chol dissolved in the bilayer and that forming the CBDs are certainly involved in the inhibitory process. For the POPC bilayer, complete inhibition of α-crystallin binding occurs before Chol reaches its saturation limit. For the SM bilayer, the presence of CBDs is necessary to fully inhibit α-crystallin binding. For the POPS bilayer, the presence of CBDs does not completely prevent α-crystallin binding.

### Association of α-Crystallin With Models of Human and Animal Lens Lipid Membranes

Experiments on mixed PL-Chol bilayers demonstrated that Chol significantly inhibits the binding of α-crystallin to these bilayers. These experiments were extended to include lipid bilayers made from the total lipid extracts of fiber cell membranes, LLMs (introduced in Section 2.2). Because LLMs are already saturated or even oversaturated with Chol,^104^^,^^134^ modification of Chol content in these bilayers is biologically challenging.

A partial solution to this problem involved substituting LLMs with their models comprising commercially available lipids with the same compositions and proportions of lipids as those in natural extracts.^48^^,^^50^ In these experiments, the binding of α-crystallin to models of the human lens-lipid (MHLL), porcine lens-lipid (MPLL), and mouse lens-lipid (MMLL), membranes with Chol content varying from 0 to 60 mol% were analyzed.^48^ The values of the MMSO and *K_a_* as a function of Chol membrane content for the three LLM models were evaluated.^48^

For models of LLMs that did not contain Chol, the highest MMSO value was for MHLL, followed by MPLL and MMLL. In contrast, the highest *K_a_* value was for MPLL, then for MHLL and MMLL. As the amount of Chol increased, both MMSO and *K_a_* decreased, but in different ways. This suggests that the PL composition of the model LLMs influences both the binding of α-crystallin and the effect of Chol on that binding. A similar pattern was also observed in single-lipid bilayers.

It is noteworthy that complete inhibition of α-crystallin binding to models of LLMs occurs when the bilayers are saturated or oversaturated with Chol. Additionally, an interesting observation is that the parameters MMSO and *K_a_* that assess α-crystallin binding to models of LLMs are not simply weighted sums of the corresponding values obtained for single-lipid bilayers composed of the lipids present in LLMs – weights reflecting the molar fraction of individual phospholipids in each LLM.^48^^–^^50^ This may suggest that LLMs possess a unique property that cannot be reduced to a simple sum of the properties of the constituent lipids.^49^

The results presented so far indicate that the binding of α-crystallin to lipid bilayers made of commercially available lipids is inhibited when these bilayers contain either saturating or oversaturating levels of Chol. One would expect that bilayers derived from natural total lipid extracts (LLMs) of intact membranes from fiber cells in the lens cortex and nucleus, which are saturated or even oversaturated with Chol,^104^ will also follow this pattern.

The results for native LLM align closely with those of model LLMs. The values for MMSO, estimated using CSL with the monitoring nitroxide group located beneath the headgroup regions for LLMs,^45^^,^^46^ are very similar to those of model LLMs^48^ and single-lipid bilayers that contain Chol.^131^

For native bovine cortical LLMs, no binding is observed (MMSO ≈ 0), although minimal binding is noted for nuclear LLMs (MMSO approximately 1%).^46^ Results obtained using 4-palmitamido-TEMPO spin label, with the monitoring nitroxide group located on the membrane surface (see Fig. 1), indicate that the binding of α-crystallin to native human LLMs increases with the severity of cortical and nuclear cataract.^44^

The data collected using the 4-palmitamido-TEMPO spin label revealed that α-crystallin preferentially binds to bovine nuclear LLMs, which have a high Chol/PL molar ratio of 1.9 rather than to bovine cortical LLMs, which have a significantly lower Chol/PL molar ratio of 0.7. Both membrane types were isolated from the eyes of a 2-year-old bovine.^47^

These findings raise the question of why α-crystallin binds to the surface LLMs despite their having high Chol content. It is suggested that this surface binding might be necessary to maintain homeostasis in lens membrane and lens cytoplasm.^46^ However, once α-crystallin binds at deeper locations within the membrane, it may create a barrier to the diffusion of antioxidants across the fiber cell membrane, potentially leading to the development of cataracts.

**Figure 6.**
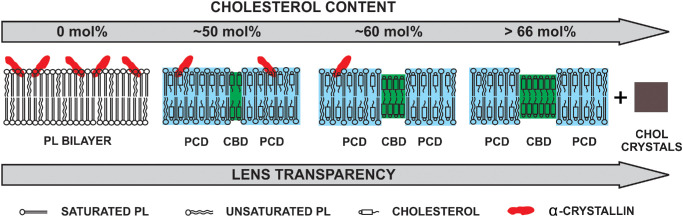
Schematic drawing showing the association of α-crystallin with the PL bilayers as a function of the increased Chol content. Chol inhibits the binding of α-crystallin to membranes. This figure is taken from Reference 131 without any modification and with permission from MDPI.

The results with no binding for bovine cortical LLM (MMSO ≈ 0) and very low binding with nuclear LLM (MMSO approximately 1%)^46^ obtained below the headgroup regions with CSL align very well with results of model LLMs and simpler lipid bilayers obtained with CSL (see Fig. 5). The studies on the binding of α-crystallin to lipid bilayers mimicking membranes of fiber cells, described in this review paper, are summarize in the schematic drawing in Figure 6.

This figure illustrates a decrease in α-crystallin binding to lipid bilayers with increased Chol content. As the Chol content rises and saturates the membrane, a PL-Chol domain is formed (PCD, shown in blue). As the Chol content continues to increase, CBDs (shown in green) begin to form within the PL-Chol domain. CBDs appear at approximately 50 mol% Chol in the PC, SM, and PS bilayers, and at approximately 33 mol% Chol in the PE bilayers.^40^ The size of the CBDs increases with increasing Chol content,^34^ and at approximately 66 mol% Chol, Chol crystals start to appear.^40^

## Congenital Cataract and Chol

The causes of congenital cataracts are diverse. It may manifest as a single hereditary characteristic, as part of a more comprehensive syndrome or illness, such as diabetes or disorders affecting the metabolism of Chol, or as the consequence of unknown causes.

Congenital cataracts are caused by gene abnormalities that fall into four major categories: mutations in genes that encode (1) crystallins, (2) lens membrane proteins like connexin and aquaporin, (3) cytoskeletal structural proteins, and (4) other genes, such as ITX3 (paired-like homeodomain 3), PAX6 (paired box 6), HSF-4 (heat shock protein factor 4), and HCR7 (7-dehydrocholesterol reductase).^135^

Due to the aqueous humor’s extremely low Chol level and the limited uptake of Chol from plasma, the adult eye lens mostly relies on de nova Chol synthesis to supply its needs for Chol. Many congenital illnesses, including Smith-Lemli Opitz syndrome, desmosterolosis, and lathosterolosis,^136^^–^^139^ exhibit cataracts as common side effects of mutations in the enzymes involved in Chol synthesis and transport. It is not yet clear to what extent these diseases are caused by the reduction in Chol function, as opposed to the effects of accumulation of precursors and byproducts. However, the importance of membrane integrity in the development and maintenance of lens homeostasis and Chol biosynthesis in prevention of cataractogenesis is shown by the prevalence of congenital and juvenile cataracts in systemic Chol synthesis disorders.

### Chol Reverses Development of Lens Defects in Zebrafish Genetic Model

Zebrafish are an important animal model for study of congenital cataracts due to the genetic and anatomic similarities with the human eye.^140^ Abundant genetic mutant lines are available to study loss-of-function alleles, and novel editing can be carried out to inactivate or otherwise edit genes of interest as desired. During their evolutionary history, the zebrafish genome has been completely duplicated; although many of these gene duplicates were rapidly lost, there often are two (or more) zebrafish orthologs of genes that are present in a single copy in humans or in mammalian animal models like mice. Human lenses express two crystallin proteins: αA-crystallin is specific to the lens only and is associated with cataracts when mutated, whereas αB-crystallin is more broadly expressed and can cause both cataracts and cardiomyopathies when mutated.^141^^–^^143^ In the zebrafish lens, mutations in αB-crystallin (*cryaba*), whose expression only becomes activated in the lens after 10 days post-fertilization, lead to lens opacities, whereas mutations in αA-crystallin (*cryaa*) show no gross changes in lens transparency.^53^^,^^144^ It appears that the protective chaperone role for the *cryaba* gene product is most important for maintaining lens clarity as the zebrafish ages. Our experimental work, mainly on model systems, indicated that high Chol is one factor – perhaps the most significant – that protects human lenses against development of age-related cataracts. This hypothesis was confirmed by work on a zebrafish mutant model with inactivated αB-crystallin (*cryaba–/–*). This model is relevant because *cryaba* mutation causes lens defects to manifest as congenital cataracts with increased lens light scattering.^53^ Both humans and zebrafish develop cataract formation as they age, which is caused by protein aggregation that impairs lens clarity. On the other hand, congenital cataracts have been linked to a variety of genes and are caused by disruptions in lens formation. The most interesting observation from this zebrafish genetic model is that further stressing the zebrafish by also mutating Nrf2 (*nfe2l2a–/–*) (i.e. the master oxidative stress factor) reduced lens defects.^54^ Transcriptional analysis showed that, in this *cryaba–/–* *nfe2l2a–/–* double-mutant model, several Chol biogenesis genes were upregulated. When statins were administrated to this double-mutant model, eye lens defects and cataracts were observed again. All these factors strongly suggest the involvement of Chol, especially in protecting against age-related cataract development. The above discussion on results obtained from the zebrafish in vivo model remains speculative. In our future experiments, Chol will be directly measured for each mutant, which will strengthen our conclusions through experimental testing.

**Figure 7.**
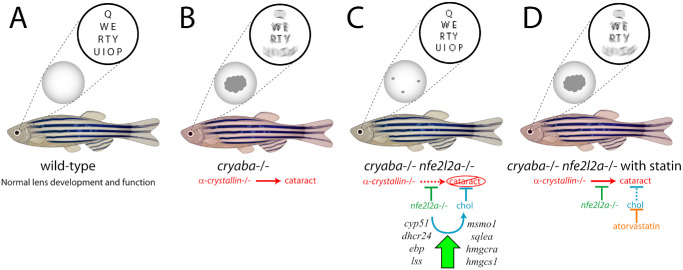
Schematic drawing of zebrafish lenses and their prevention or causation of cataract in response to genetic and external factors. (**A**) Wild-type zebrafish have normal lens development with no cataracts expected. (**B**) Loss of αB-crystallin (*cryaba*) leads to the frequent presence of cataracts and loss of clear vision. (**C**) Loss of both *cryaba* and Nrf2 (*nfe2l2a*) reduces frequency and severity of cataracts due to upregulation of Chol synthesis factors. (**D**) Treatment of *cryaba–/–* *nfe2l2a–/–* mutants with atorvastatin reduces Chol levels, allowing cataracts to become prevalent again in this genetic background.

The need to reduce oxidative stress is a phenomenon common to different species and tissue types owing to the destructive potential of this process. Central to antioxidant mechanism and its regulation is the transcription factor Nrf2. In response to oxidative stress cues, Nrf2 translocates to the cell nucleus to activate genes whose function is to reduce this stress, including members of glutathione and thioredoxin metabolism, NADPH production, multidrug resistance, and more. The long-lived lens must maintain constant clarity throughout life, and this can be threatened by cataract formation initiated by oxidative stress. The lens relies on antioxidant defenses, including glutathione, to maintain transparency against protein aggregation from oxidation.^145^ Experiments conducted to study the loss of zebrafish Nrf2 (*nfe2l2a*) in combination with loss of αB-crystallin revealed intriguing but initially counter-intuitive findings: the loss of *nfe2l2a* activity reduced the appearance of cataracts, even though the role of Nrf2is to counteract oxidative stress.^54^ Further investigation revealed that although loss of Nrf2 function likely leads to an inability to upregulate glutathione using this mechanism, the lenses still have fewer cataracts. The reasons for this are discussed below and illustrated in Figure 7.

Park and colleagues combined *nfe2l2a* and *cryaba* mutant lines to study the effects of loss of Nrf2 function on cataract formation in crystallin-deficient lenses.^54^ Although the role of Nrf2 is to provide antioxidant support for at-risk tissues and cells, cataracts were paradoxically absent from *nfe2l2a/cryaba* double mutants (see Fig. 7C). It was found that genes involved in Chol biosynthesis – *cyp51*, *dhcr24*, *ebp*, *lss*, *msmo1*, *sqlea*, *hmgcra*, and *hmgcs1* – were upregulated in *nfe2l2a* mutants, which appeared to protect the lens from cataracts. This experimental finding is key to the hypothesis that increased levels of Chol act to block cataract formation in the eye. This was confirmed by the application of atorvastatin to reduce Chol levels, resulting in the reappearance of cataracts in the *nfe2l2a cryaba* double mutants (see Fig. 7D). Future work will examine the lipid composition of wild-type zebrafish lenses, as well as *cryaba* single mutants, *nfe2l2a* single mutants, and *nfe2l2a cryaba* double mutants. Understanding the lipid composition of the lenses from these mutant lines will help to develop therapeutic treatments to reduce or prevent cataracts in human patients.

### Association Between Chol Deficiency and Cataract Formation

The timing of cataract onset is determined by whether it results from a detrimental genetic mutation or from accumulated biomolecular damage. Several disorders involving enzyme defects in post-squalene Chol biosynthesis have been identified and are linked with the development of congenital cataracts: desmosterolosis, CHILD syndrome, lathosterolosis, X-linked dominant chondroplasia punctata 2, and Smith–Lemli–Opitz syndrome.^136^^,^^146^^,^^147^ The most common Chol biosynthetic disorder is Smith-Lemli-Opitz syndrome, whose underlying defect was identified in 1964.^148^ Reduced or absent 7-dehydrocholesterol reductase enzyme (DHCR7), which catalyzes the stage of Chol production, is either lacking or exhibits insufficiency in this syndrome. This results not only in Chol deficiency but also in accumulation of 7-dehydrocholesterol, 8-dehydrocholesterol, and toxic oxysterol metabolites. The cause of cataracts in this disease was postulated to be due to the decrease in Chol level in the lens.^149^ Following the identification of the biochemical flaw that causes Smith-Lemli-Opitz syndrome, a diagnostic test and a potentially helpful treatment (dietary Chol supplementation) have been developed. Using positional cloning, Masayuki Mori et al.^149^ tried to determine the genes linked to cataract development in Shumiya cataract rats. A particular gene deficit combination was linked to cataract development, lowering Chol levels in cataractous lenses to roughly 57% of normal. The researchers think their discoveries might inform on congenital problems of Chol synthesis and the impact of Chol-lowering drugs on other forms of cataracts. Their research supports the hypothesis that Chol deficiency alone may induce cataracts similar to our group's preliminary data suggesting that increased synthesis of Chol reverses development of lens cataracts in the zebrafish genetic model.

In animal studies, compounds that have the ability to block the biosynthesis of Chol appear to cause lens damage.^150^^–^^152^ Vries et al. observed different effects between two statin drugs (simvastatin and pravastatin) in the ability to affect the Chol content of the lens in vivo.^151^ Simvastatin was found to be 100-fold more effective at inhibiting Chol synthesis in rat lenses than pravastatin. Rats given simvastatin (10 mg/kg body weight) in their food saw lens Chol levels drop by more than 25% after 3 weeks, as compared with a control group.^151^ The study by Gesron et al. revealed that administering high doses of various hydroxymethylglutaryl-CoA reductase inhibitors led to the formation of subcapsular lenticular opacities in dogs.^152^ However, no changes in Chol content were observed in the lenses of dogs given a chronic dose of HMG-CoA reductase inhibitors. In contrast, another drug that blocks Chol transport, the oxidosqualene cyclase inhibitor U18666A, was found to be highly likely to cause cataracts in animals.^153^ Research has shown that the cataracts caused by U18666A are linked to a selective decrease in the levels of γ-crystallin. Mitchell and Cenedella^154^ based their studies on human lenses on the hypothesis that high Chol is necessary for lens transparency and examined the impact of Chol-lowering drugs on crystalline lenses. The Chol concentration in patients aged 60 to 67 years who had been taking lovastatin or simvastatin before their death was not found to be different from the control group.^154^ Our lab’s research has shown that reduction of Chol biosynthesis may cause lens opacity. However, it should be mentioned in this context that a number of factors, including reduction of the elongation rate or the differentiation process of lens fibers, change in Ca^2+^ concentration, osmotic stress induction, and chloride channel blockage, may also cause lens opacity. Some drugs have been shown to have no effect on the amount of Chol in the lens, whereas others have been shown to significantly reduce the accumulation of Chol.^151^ It must finally be stressed that the overall effect of Chol-lowering drugs on lens health is complex and debated and studies, especially clinical are not conclusive.^155^^–^^158^ Some clinical studies have suggested an increased risk of cataracts, particularly among long-term users of high-dose statins. Others have found no significant association. A few have even hinted that statins might reduce cataract risk due to their anti-inflammatory and antioxidant properties, which help to protect the eyes. Thus, the pathogenesis of lens opacity associated with disruption of Chol homeostasis still needs more studies.

## Concluding Remarks

This review paper proposes the hypothesis that Chol, known to be important for lens homeostasis, is one of several potential factors in protecting human lenses from developing age-related cataracts, and may be the most significant, or key factor. As stated in the introduction, our objective is to demonstrate how Chol may counteract the degenerative processes occurring in the eye lens throughout a person's lifespan, ultimately leading to cataracts and impaired vision. Sections 2 and 3 describe the studies on model membranes (lipid bilayers) that mimic the membranes of lens fiber cells and intact membranes. These studies infer that Chol can protect the human eye lens from these degenerative processes and preserve clear vision. Section 4 presents the in vivo whole animal zebrafish mutant model studies, and shows that Chol inhibition or upregulation is associated with improvement or deterioration in the transparency of the zebrafish lenses.

In Section 2, we focus on the biophysical studies of Chol in model and intact membranes of fiber cells. Immature fiber cells, composed of membrane and cytoskeleton, are the only supramolecular structures of the cell. All of the Chol within the cell is accumulated in the membrane,^124^^,^^159^^–^^161^ except for Chol crystals that detach from the membrane and become kinetically frozen, thus not participating in Chol-related activities. The Chol level in these membranes is at least saturating. This saturation ensures that the physicochemical properties of the membrane remain constant, regardless of any age-related changes in the membrane PL composition, thereby ensuring the proper functioning of membrane proteins. Consequently, membrane homeostasis is maintained.^124^^,^^159^^–^^161^

As indicated through this review, the lipid environment can affect membrane protein function. However, lens lipid changes associated with age and cataract formation have not been yet extensively examined with spatial resolution. Recently, Schey and colleagues presented findings of lens lipid changes with age and cataract in human lenses using imaging mass spectrometry and spatially-resolved lipidomics analysis.^162^ They reported the detection of large gangliosides (GM1 and GM3) in lens cortical fiber cells and smaller lactosylceramides (GA1 and GA2) in lens nuclear fiber cells. The identification of modified lipids and Chol species in transparent and cataractous human lenses associated with aging is timely and needs further research.

In Section 3, the study on the effect of Chol on the binding of α-crystallin to model membranes is described. The α-crystallin is a cytoplasmic protein that in its soluble, polydispersed oligomeric form helps maintain lens transparency.^128^ However, as humans age, the water-soluble fraction of α-crystallin decreases while the membrane-bound fraction increases.^6^^,^^8^^,^^11^^,^^12^^,^^43^^,^^44^ This leads to light scattering and lens opacification.^6^^,^^8^^,^^11^ The results of model studies indicate that a high level of Chol in the membrane inhibits the binding of α-crystallin, which in turn reduces light scattering. This suggests that the high Chol content in the membranes of fiber cells may play a similar protective role, helping to prevent lens opacity.

Section 4 details studies conducted in vivo using a zebrafish mutant model.^53^^,^^144^ This is significant because the previous sections describe only works involving model bilayer membranes. The zebrafish mutant with inactivated α-crystallin causes lens defects that manifest as congenital cataracts with increased lens light scattering. This model illustrates that further genetic modification, through the upregulation of a number of genes responsible for Chol biogenesis, reduced lens defects.^54^ Thus, the results with the double α-crystallin/ Nrf2 (*cryaba/ nfe2l2a*) mutant model clearly show that Chol is not only involved in these processes but can reverse cataracts that have already developed. When statins are introduced to the zebrafish double mutant model, the cataracts return.^54^ This research is in progress, and experimental determination has not yet concluded. We are planning to measure Chol levels in each zebrafish genetic model. This will experimentally test our hypothesis that the key function of Chol in the lens is to prevent lens cataract.

Within the last year, numerous papers were published showing that in vivo manipulation of lens Chol can reverse cataracts that have already developed. These data, to a certain degree, support our statement based on zebrafish mutant model. We acknowledge the contradictory results that the Chol precursor lanosterol shows evidence of clearing cataracts in some studies, but not in others, including work by Daszynski et al.^163^ We note that Daszynski and colleagues used an acute pressure-induced form of cataractous damage, while other publications,^164^^–^^168^ including the landmark study by Zhao et al.,^168^ used lanosterol treatment on naturally occurring cataracts, which grow over time and in response to natural cues. Lanosterol and 25-hydroxycholesterol may have better anti-cataractous properties on natural and spontaneous cataracts, than those induced by UV light exposure, as published.^168^ The combination of nilvadipine with lanosterol has been shown to help in cases of selenite-induced cataractic rats.^164^ Finally, ongoing work with 25-hydroxycholesterol treatment (commercialized as VP1-001),^166^ an oxysterol,^167^ continues to study the abilities of Chol derivatives to clear cataracts by binding to crystallins in the lens.

The studies and results presented in this review paper strongly support the hypothesis that a very high Chol content in the membranes of fiber cells plays a crucial role in protecting the eye lens. This protection likely occurs both at the membrane and cellular levels, ensuring the transparency of the eye lens. This role of Chol has been evolved throughout the biological evolution of the simple camera eye lens, to enable it to properly focus light onto the retina and maintain clear vision. At the membrane level, the high Chol content makes the physical properties of the membrane resistant to changes in the membrane PL composition, which is necessary for the long-term functioning of membrane proteins. At the cellular level, the high Chol content in the membrane inhibits the binding of α-crystallin, which reduces light scattering and prevents lens opacity.^6^^,^^8^ Furthermore, and most significantly, Chol has the ability to reverse the development of cataracts.^53^

The studies presented here shed light on the largely unexplored functions and mechanisms of Chol action in both healthy and cataractous lenses. We believe that these hypotheses may help identify new strategies for preventing, slowing down, or even reversing the age-related development of cataracts, which is essential for maintaining lens transparency.

## Supplementary Material

Supplement 1
