# Supplementary material for: Preventing Age-Related Nuclear Cataract Development – Is Cholesterol the Key?
Source: Invest Ophthalmol Vis Sci. 2026 Jan 15;67(1):34. doi: 10.1167/iovs.67.1.34 (PMC12814978; doi:10.1167/iovs.67.1.34)
Supplement: Supplement 1 [file iovs-67-1-34_s001.docx]

**Supplementary Materials**

**Introduction**

To clearly understand the information presented in transmembrane profiles in Figs. 2, 3, and 4 we briefly describe the methods by which they were obtained and explain what measured parameters indicated in terms of membrane structure and dynamics. Lipid spin labels with a small monitoring group, a nitroxide moiety, and that were rigidly attached at a certain position to the parent phospholipid or cholesterol molecule (see Fig. 1) allowed the monitoring moiety to be put into the particular bilayer phase or domain and at the certain depth in the bilayer. With the electron paramagnetic resonance (EPR) techniques, two classes of parameters can be obtained, describing nondynamic (profiles of the order parameter and hydrophobicity) and dynamic (profiles of spin-lattice relaxation rate [*T*_1_^-1^] and oxygen transport parameter [OTP]) membrane properties. Profiles of the order parameter and spin-lattice relaxation rate describe the property of the fragment of the parent PL and Chol molecules to which the nitroxide monitoring group is rigidly attached. Profiles of the oxygen transport parameter and hydrophobicity describe property of the local microenvironment of the nitroxide moiety, and we call them physicochemical membrane properties. Information about the structure and dynamics of complex model and biological membranes can be obtained using lipid spin labels that mimic the behavior of the parent molecules (PLs and Chol).

**Order Parameter**

In fluid phase lipid bilayers, the acyl chain of the PL molecule undergoes a rapid unconstrained rotation about its long axis and a wobbling motion of this long axis within the confine of a cone imposed by the membrane environment. With the increasing cone angle, the value of the order parameter decreases, providing an order parameter profile along the acyl chain. The order parameter can be obtained directly from the EPR spectra using procedure described in Ref.^1^. The order parameters of PL acyl chains in the bilayer can be obtained from EPR spectra for one-phase regions (as in Figs. 2B and 3A). In the lens lipid membranes, in which CBDs are already present, the order parameter reflects the order of the acyl chains in the Chol-saturated PL bilayer surrounding the CBDs because the PL-analog spin labels do not partition into CBDs.

For intact biological membranes, with coexisting membrane domains, the order parameter is averaged over spin labels located in these coexisting domains. Additionally, because of the overlapping components, the EPR spectra do not contain features needed for the calculation of the order parameter. In that case, we are using the maximum splitting (i.e., the distance between the outermost peaks of the EPR spectrum as a measure of membrane order (Fig. 4A). For the fast anisotropic motion of spin labels the maximum splitting is directly related to the order parameter^2^ that indicates the amplitude of the wobbling motion of the appropriate segment of the alkyl chain of n-SASL^3^. This approach sometimes allows us to obtain values of the maximum splitting of two components from the EPR spectra, as in the case presented in Fig. 4A. Discrimination of the domains depends on the spin label exchange rate between domains, which differently affects the measured parameters. As shown in the profiles in Fig. 4A, the maximum splitting parameter can discriminate the bulk plus boundary domain from the trapped lipid domain.

Splitting values between appropriate peaks in the PL spin labels used for the calculation of the order parameter (as in Figs. 2B and 3A) were measured within the precision of ±0.25 G. Maximum splitting values for intact membranes (as in Fig. 4A) were measured within the precision of ±0.5 G and ±1.0 G for fluid and immobilized components, respectively.

**Spin-Lattice Relaxation Rate (*T*_1_^-1^)**

A computer simulation of the EPR spectra of n-PC and n-SASL using a program based on Freed's microscopic-order macroscopic-disorder model^4^ can provide the rotational diffusion coefficient for these spin labels and, thus, dynamic parameters. This procedure is difficult and time consuming. Fortunately, the spin-lattice relaxation rate (*T*_1_^-1^) of PL-analog spin labels obtained from saturation recovery (SR) EPR measurements in the absence of other paramagnetic molecules such as O_2_ (for deoxygenated samples) is determined primarily by the rotational diffusion of the nitroxide moiety ^5-7^. Profiles of *T*_1_^-1^values can be easily obtained and presented (Figs. 2C, 3B, 4B). A larger *T*_1_^-1^value indicates faster rotation and consequently higher membrane fluidity. Additionally, calibration curves can be created that connect the *T*_1_^-1^ values with the coefficient of the rotational diffusion of the specific monitoring nitroxide moiety attached to the PL-analog spin labels^8^. However, for convenience (in Figs. 2C, 3B, 4B) in our presentations, we used basic profiles of *T*_1_^-1^ to report effects of membrane composition on the membrane fluidity.

The uncertainties in the measurements of decay time (*T*_1_) from the fits were usually less than 0.05%, whereas the decay times determined from sample to sample were within a precision of ±3% when a single-exponential fit was satisfactory and within a precision ±5% and ±10% for longer and shorter recovery time constants when a double-exponential fit was satisfactory.

**Oxygen Transport Parameter (OTP)**

Bimolecular collisions of a nitroxide moiety of the spin label with molecular oxygen induce spin exchange, enhancing spin-lattice relaxation of the nitroxide. This effect can be measured directly using SR EPR. Kusumi et al. introduced a convenient parameter named the OTP^9^ to evaluate the rate of collisions between spin labels and O_2_:

OTP(*x*) = *T*_1_^-1^(Air, *x*) − *T*_1_^-1^(N_2_, *x*) (S1)

Here, *T*_1_^-1^s are the spin-lattice relaxation rates of the nitroxide moiety of the spin label positioned at the depth *x* (distance from the membrane center) of the membrane equilibrated with air (Air) and nitrogen (N_2_). Thus, to get the value of the OTP, two SR EPR signals have to be measured, one of a deoxygenated sample and the other of that equilibrated with air. The OTP can also be expressed by the Smoluchowski equation, which takes the following form:

OTP(*x*) = *AD*_SL_(*x*) + *D*(*x*)*C*(*x*), *A* = 4π*pr*_o_ (S2)

Here, *D*(*x*) and *C*(*x*) are, respectively, the O_2_ diffusion coefficient and the O_2_ concentration at the depth *x* (around the nitroxide moiety) in the membrane equilibrated with air; *D*_SL_(*x*) is the diffusion coefficient of the lipid spin label; *r*_o_ the interaction distance between O_2_ and the nitroxide moiety of the spin label, equal to 4.5 Å^10^; and *p* is the probability that a spectroscopically observable event occurs when a collision takes place. In the SR EPR methodology, *p* is assumed to be 1. Since the *D*(*x*) (diffusion coefficient of O_2_) in membranes is much greater than the *D*_SL_(*x*) (diffusion coefficient of the lipid spin label), the latter can be omitted in Eq. S2, which now takes a simpler form:

OTP(*x*) = *AD*(*x*)*C*(*x*) (S3)

The combination of Eq. S1 and Eq. S3 provides the method of obtaining the oxygen diffusion-concentration product, *D*(*x*)*C*(*x*), from the SR EPR measurements. It should be stressed here that when using experimental methods, it is not possible to factor this product into *D*(*x*) and *C*(*x*). It was shown that *A* is independent of the kind of a spin label, local hydrophobicity, and local viscosity of the environment around the nitroxide moiety. The profiles of the OTP (and thus the oxygen diffusion-concentration product) are shown in Figs. 2D, 3C, and 4C. We can state that the OTP is a useful monitor of membrane fluidity that reports on translational diffusion of small molecules.

**Hydrophobicity of Membrane Interior (2*A*_Z_)**

The membrane interior becomes increasingly more hydrophobic (nonpolar) from the membrane surface to the membrane center. This hydrophobicity gradient is largely determined by the degree of water penetration. The EPR spin-labeling method of assessing membrane hydrophobicity is based on the fact that an increase in the solvent polarity increases the *z* component of the hyperfine interaction (measured as the maximum splitting in the EPR spectrum of a spin label frozen in membrane suspension), *A_Z_*. With an increase in solvent polarity, the *z* component of the hyperfine interaction, *A*_Z_, increases. In membranes (as shown in hydrophobicity profiles in Figs. 2E, 3D, and 4D), this parameter is measured as a maximum splitting (2*A*_Z_).

To give a physical meaning to the hydrophobicity measured as 2*A*_Z_, these values for selected depths in the membrane are related to the polarity (or dielectric constant, ε) of bulk organic solvents in which 2*A*_Z_ of the dissolved spin label were similar, as shown in Fig. 2 in Ref. ^11^. Incorporation of saturating amount of Chol into phospholipid bilayers (Figs. 2E and 3D) decreases polarity (increases hydrophobicity) in the middle of the bilayer from that of 2-pentanol or 1-octanol (ε = 10÷20) to the level of hexane (ε = 2). Here, we would like to stress that, in the PL bilayer not containing Chol, the hydrophobicity of the membrane interior depends on PL species, especially on PL unsaturation. Saturated bilayers do not form high hydrophobic barriers; the hydrophobicity in their center is close to that of 2-pentanol or 1-octanol (ε = 10÷20) while that in the center of unsaturated bilayers is close to that of 1-decanol (ε = 10, POPC). All these differences are canceled at saturating Chol contents because, for all bilayers, the hydrophobicity in the bilayer center is close to that of hexane (ε = 2).

To measure hydrophobicity, the *z*-component of the hyperfine interaction tensor of PL spin labels, *A*_Z_, was determined from the EPR spectra for samples frozen at −165°C within the precision of ±1.0 G.

**Changes in the Chol Saturation Limits and Chol Solubility Thresholds as a Function of Age**

To explain the mechanisms that keep fiber cell membrane properties so independent of the age of donors, and independent of the drastic differences in their PL composition, we showed the relationship between the Chol saturation limits and the Chol solubility thresholds in fiber cell membranes as a function of age of donors (Fig. S1). The data obtained with the application of both EPR and DSC methods allowed us to obtain not only the appropriate values of the Chol solubility thresholds but also the values of the Chol saturation limits for mixtures of major lens PLs—namely, PC, PS, PE, and SM—with Chol, presented in Ref.^12^. The obtained values of the Chol saturation limit are 33 mol%, 50 mol%, 50 mol%, and 50 mol% Chol in the PE, PC, PS, and SM bilayers, respectively, and the obtained values of the Chol solubility threshold are 50 mol%, 66 mol%, 66 mol%, and 66 mol% Chol in the PE, PC, PS, and SM bilayers, respectively ^12^. To calculate the values of the Chol saturation limits and Chol solubility thresholds presented in Fig. S1 we assumed, similarly as in Ref. ^12^, that these values in PL mixtures are the weighted sums of the Chol saturation limits and Chol solubility thresholds for individual PLs with a weight equal to the mole fraction of the individual PLs in the mixture taken from Ref. ^12^.

As shown in Fig. S1, the values of the Chol saturation limits and Chol solubility thresholds practically do not change with the donor age, independently of the drastic changes in their PL composition. From the date of the individual PLs, it follows that only PE can decrease the Chol saturation limits and Chol solubility thresholds in PL mixtures. The PE content in fiber cell membranes of different ages is small and changes from 26 mol% for 13-year-old donors to 12 mol% for 70-year-old donors^13^. This explains why the evaluated Chol saturation limits and Chol solubility thresholds are independent of the donor’s age and why their values are only a little scattered (Fig. S1).


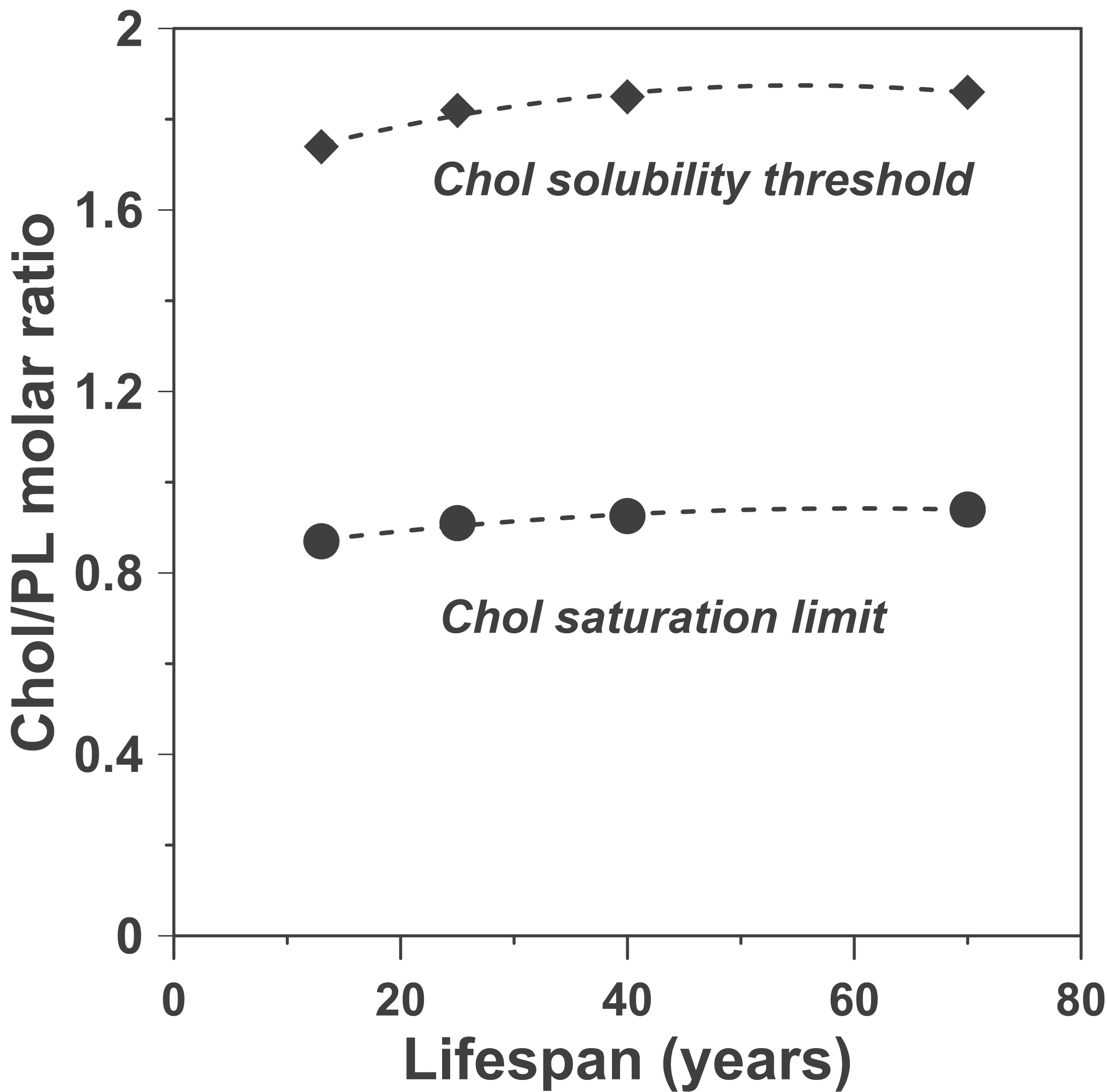


Fig.S1. The relationship between the Chol saturation limit (●) and the Chol solubility threshold (♦) in the human fiber cell membranes and the age of the donor. CBDs should be formed at Chol concentrations greater than the Chol saturation limits. Chol crystals should be formed at Chol concentrations greater than the Chol solubility thresholds. Chol saturation limits and Chol solubility thresholds were evaluated based on the PL compositions taken fromRef.^13^ and the phase diagram presented in Ref.^12^. Points are for human donors aged 13, 25, 40, and 70 years.

**References**

1. Marsh D. Electron Spin Resonance: Spin Labels. In: Grell E, ed. *Membrane Spectroscopy*. Berlin, Heidelberg: Springer Berlin Heidelberg; 1981:51-142.

2. Hubbell WL, McConnell HM. Spin-label studies of the excitable membranes of nerve and muscle. *Proc Natl Acad Sci U S A*. Sep 1968;61(1):12-16. doi:10.1073/pnas.61.1.12

3. Kusumi A, Subczynski WK, Pasenkiewicz-Gierula M, Hyde JS, Merkle H. Spin-label studies on phosphatidylcholine-cholesterol membranes: effects of alkyl chain length and unsaturation in the fluid phase. *Biochim Biophys Acta*. Jan 29 1986;854(2):307-317. doi:10.1016/0005-2736(86)90124-0

4. Meirovitch E, Freed JH. Analysis of slow-motional electron spin resonance spectra in smectic phases in terms of molecular configuration, intermolecular interactions, and dynamics. *The Journal of Physical Chemistry*. 1984/10/01 1984;88(21):4995-5004. doi:10.1021/j150665a041

5. Robinson BH, Haas DA, Mailer C. Molecular dynamics in liquids: spin-lattice relaxation of nitroxide spin labels. *Science*. Jan 28 1994;263(5146):490-493. doi:10.1126/science.8290958

6. Mailer C, Nielsen RD, Robinson BH. Explanation of Spin−Lattice Relaxation Rates of Spin Labels Obtained with Multifrequency Saturation Recovery EPR. *The Journal of Physical Chemistry A*. 2005/05/01 2005;109(18):4049-4061. doi:10.1021/jp044671l

7. Marsh D. Molecular order and T1-relaxation, cross-relaxation in nitroxide spin labels. *Journal of Magnetic Resonance*. 2018/05/01/ 2018;290:38-45. doi:

8. Subczynski WK, Widomska J. Spin-Lattice Relaxation Rates of Lipid Spin Labels as a Measure of Their Rotational Diffusion Rates in Lipid Bilayer Membranes. *Membranes (Basel)*. Sep 30 2022;12(10)doi:10.3390/membranes12100962

9. Kusumi A, Subczynski WK, Hyde JS. Oxygen transport parameter in membranes as deduced by saturation recovery measurements of spin-lattice relaxation times of spin labels. *Proc Natl Acad Sci U S A*. Mar 1982;79(6):1854-1858. doi:10.1073/pnas.79.6.1854

10. Windrem DA, Plachy WZ. The diffusion-solubility of oxygen in lipid bilayers. *Biochim Biophys Acta*. Aug 14 1980;600(3):655-665. doi:10.1016/0005-2736(80)90469-1

11. Subczynski WK, Wisniewska A, Yin J-J, Hyde JS, Kusumi A. Hydrophobic Barriers of Lipid Bilayer Membranes Formed by Reduction of Water Penetration by Alkyl Chain Unsaturation and Cholesterol. *Biochemistry*. 1994/06/21 1994;33(24):7670-7681. doi:10.1021/bi00190a022

12. Mainali L, Pasenkiewicz-Gierula M, Subczynski WK. Formation of cholesterol Bilayer Domains Precedes Formation of Cholesterol Crystals in Membranes Made of the Major Phospholipids of Human Eye Lens Fiber Cell Plasma Membranes. *Current eye research*. 2020;45(2):162-172. doi:10.1080/02713683.2019.1662058

13. Huang L, Grami V, Marrero Y, et al. Human lens phospholipid changes with age and cataract. *Invest Ophthalmol Vis Sci*. May 2005;46(5):1682-1689. doi:10.1167/iovs.04-1155
